# Nothing to Cheer About: Endorsing  Imaginary Economic Evaluations and Value Claims with CHEERS 22

**DOI:** 10.12688/f1000research.109389.1

**Published:** 2022-02-28

**Authors:** Paul Langley

**Affiliations:** 1 College of Pharmacy, University of Minnesota, Minneapolis, MN, 55455, USA

**Keywords:** CHEERS 22, ISPOR, ICER, pseudoscience, I-QALY

## Abstract

One of the more unfortunate features of health technology assessment is the tenacity with which leaders in the field and organizations such as the International Society for Pharmacoeconomics and Outcomes Research (ISPOR) and the Institute for Clinical and Economic Review (ICER) cling to an evaluation framework that fails to meet the standards of normal science. Believers subscribe to a meme that is clearly non-science (metaphysics and pseudoscience) and one that should have been discarded over 30 years ago. Certainly, subscribing to an impossible belief is not unusual; indeed it may make the belief that much stronger. Yet the meme is non-sustainable; it is also pointless as the economic evaluation claims are non-evaluable. There is no acknowledgement of the standards of normal science or the limitations imposed by the axioms of fundamental measurement. The purpose of this commentary is to make the case that the recent release of the Consolidated Health Economic Evaluation Reporting Standards 2022 (CHEERS 22) checklist is misleading; CHEERS 22 fails to address the manifest deficiencies in the approach to economic evaluations endorsed by ISPOR and ICER. Instead, it continues to promote economic evaluations in healthcare that invent evidence and non-empirically evaluable value claims. Given the widespread publicity that has accompanied the release of CHEERS 22, the purpose of this commentary is to detail the deficiencies in CHEERS 22 and propose an alternative framework for economic evaluation in health care to meet the information needs of formulary committees. This means abandoning the standards for economic evaluations that have dominated health technology assessment for 30 years, notably the key role assigned to the mathematically impossible quality adjusted life year (QALY). The proposed new start recommends single attribute evaluable value claims that meet ratio or interval measurement standards and are supported by evaluation protocols.

## Introduction

Concerns that economic evaluations in health technology assessment are misguided are not new; over the past 15 years there have been numerous commentaries on the manifest failures of assumption driven simulations advocated by, among others, the International Society for Pharmacoeconomics and Outcomes Research (ISPOR) and the Institute for Clinical and Economic Review (ICER) in the US. ^1^^–^^4^ Central to these critiques has been the disregard by ISPOR/ICER of the standards of normal science where value claims must be credible, evaluable and replicable. ^5^ Add to this disregard of the standards of normal science is the mathematically impossible quality adjusted life year (QALY); ignoring the fact that the multiattribute preference scores are simply composite ordinal scores rather that the single attribute bounded ratio measure that you require to construct a QALY. ^6^ The preference scores in turn lack dimensional homogeneity and construct validity. This neglect of fundamental measurement results in assumption driven simulations that generate invented claims as well as denying simple logic, with the result that formulary committees and other health system decision makers are faced with the possibility of multiple competing assumption driven imaginary claims in disease areas, which seems a pointless exercise. ^7^

Unfortunately, inventing evidence through modeled simulations continues. This commentary takes the position that as this invention of evidence is the dominant meme in health technology assessment it has little to offer decision makers in health care systems. ^8^ This is seen in the recent release of the Consolidated Health Economic Evaluation Reporting Standards 2022 (CHEERS 22) checklist as guidance for presenting imaginary economic evaluations to academic journals. ^9^^,^^10^ The purpose of this commentary is to make the case that the CHEERS 22 guidance is redundant; it is an analytical dead end. The case to be presented is for abandoning the approximate information economic evaluation meme in health technology assessment, in favor of a new start where, as this commentary argues, value claims for pharmaceutical products should be in terms of single evaluable core value attributes supported by assessment protocols. ^11^

A critical review and request for withdrawal of the CHEERS 22 guidance for economic evaluations is made that more pressing by the fact that the guidance is a joint publication of some 15 international journals, with the peer review for these journals being undertaken by the British Medical Journal (BMJ). While infeasible, one might hope for retractions by each journal; unless, of course, as this commentary points out, they are willing to accept imaginary claims for competing pharmaceutical products as an essential part of their editorial policy for the foreseeable future.

### Paradigms and memes

It is important, given the focus here on the standards of normal science, to explain why the approximate information belief of ISPOR/ICER and CHEERS 22 should be seen as a meme rather than a paradigm. Following Kuhn, a paradigm is a set of evidence-based concepts and practices, a discipline involving problems and solutions for a community. ^12^ The proposition is that sciences go through alternating periods of ‘normal puzzle solving’ and revolution where the concept of reality may experience a major change. As such, a paradigm is founded on a belief in evidence and hypothesis testing, a frame of reference consistent with the 17th century scientific revolution: epitomized by the motto of the Royal Society (1660) *nullius in verba* (take nobody’s word for it). A belief system diametrically opposed to the approximate information meme in health technology assessment where we are asked to take anybody’s word for it once it meets CHEERS 22 evidence standards.

To be clear, the approximate information belief system of CHEERS 22 is not a paradigm. It fails the simplest test: any economic evaluation of therapy impact must be expressed in empirically evaluable terms, ideally supported by a claim assessment protocol detailing how is to be evaluated and reported to a formulary committee. Although a paradigm could be described as a meme articulating a scientific doctrine, there is a fundamental difference: the ISPOR/ICER meme is a belief system that embodies faith in assumption driven constructs where truth is consensus. Demonstrating that beliefs are false may have no impact on those who subscribe to a meme; as evidenced by the continuing belief by ISPOR/ICER in inventing claims from non-evaluable assumption driven simulations and a refusal to recognize the limitations imposed by the axioms of fundamental measurement; now reinforced by CHEERS 22. Demonstrating to believers that the meme rejects the standards of normal science is immaterial; faith in the construction of imaginary simulations persists. An apt description is a belief supporting over the years a conviction that owes nothing to evidence or any notion of the scientific method; a conviction that believers feel is compelling and convincing. ^13^ With high transmission fidelity the ISPOR/ICER meme becomes self-sustaining and self-referential; *once it is believed, it automatically undermines opposition to itself*. ^11^ A Kuhnian paradigm can be rejected because it fails to resolve empirical questions that a new paradigm resolves, often incorporating principal elements of the prior paradigm. The meme admits of no such flexibility and no concept of discovery and progress; challenging the adherent’s faith invokes apostasy and even hostility as it cuts much deeper. We are asked, in CHEERS 22, to take the word of those believers who invent non-evaluable economic evaluation frameworks to support claims for cost-effectiveness.

One defense of the CHEERS 22 advocacy is to fall back on a relativist view that all perspectives in scientific inquiry are equally valid. If CHEERS 22 is viewed in a relativist framework then there is no basis for comparing the methodology and claiming superiority to the standards of normal science. It is an equally valid analytical framework. Accepting a relativist position, the CHEERS 22 belief system is to be understood sociologically; no one body of ‘evidence’ is to be judged superior to another. The success of a research program *rests on its ability to mobilize the support of the community of believers*. ^11^ Value claims are invented not discovered; assumption driven non-evaluable simulations are dominant. CHEERS 22, from this perspective, is not interested with coming to grips with reality, but in *rhetoric, persuasion and authority*. ^11^ The fatal flaw in this position is that it rejects the unique characteristic of science: *the appeal to superior evidence*. ^11^

A reasonable question is whether or not there are elements of the CHEERS 22 framework that might be acceptable. There are few minor points such as defining a target population, the role of systematic reviews and the relevance of the study question. Beyond these elements, which would characterize most studies, the CHEERS 22 guidance is focused on imaginary claims; the antithesis of what is argued for in this commentary.

### Falsification versus verification

For over 80 years following the contribution of Popper to propose falsification (or in practical terms sophisticated falsification) as the touchstone of value claims evaluation in the physical and mature social sciences has been on the discovery of new yet provisional facts through a process of conjecture and refutation. ^14^ Popper’s seminal contribution was to make clear the distinction between falsification and verification: in logic we may conclusively falsify but not conclusively verify. In practice, we may not conclusively falsify while at the same time avoid systematically falsifying claims. This still disallows the verification of claims as advocated by logical measurement (or logical positivism). As verification of imaginary assumption driven simulations is central to the ISPOR/ICER methodology of health technology assessment, this creates a major credibility problem in health care decisions. A problem made even more intractable as these lifespan ISPOR/ICER models are actually designed not to generate value claims that can be empirically evaluated. Imaginary claims, quite obviously, cannot be falsified which, to many analysts, is their appeal. It is this failure to acknowledge the standards of normal science where value claims are credible, evaluable and replicable, that forces ISPOR/ICER to fall back on verification, driven by assumption, as justification for simulation models to ‘support’ pricing and access recommendations for selected pharmaceutical products. This commitment to verification is seen in ICER simulation models where, in the case of a recent report on tirzepatide the model and its non-evaluable claims are ‘verified’ by reference to: (i) feedback from interested parties; (ii) verification of model parameters (assumptions) for face validity (realism); (iii) sharing the model with manufacturers; and (iv) comparing to revised model projections. ^15^ The same verification standards could be applied to any number of competing models to get the good housekeeping seal of approval for each model, a feature ICER of evidence reports for the last 10 years.

In proposing an asymmetry between falsification and verification, Popper offers the solution to Hume’s problem of induction which had been one of the more intractable issues in philosophy since the 18 ^th^ century, where pure empiricism (logical empiricism) is not a sufficient basis for science or universal statements, let alone progress. ^16^^,^^17^ If induction is admitted then the doors are open to claims based upon experience (face validity) or where limited objective evidence is the only foundation for knowledge. This defies simple logic: the fact that the ‘laws’ of physics have held in the past does not mean that they can be claimed to hold in the future. The fact that past futures have resembled past pasts does not mean that future futures will resemble future pasts, irrespective of the number of past observations or spurious probabilistic claims for the relevance or realism of assumptions. ^18^ Claims for the relevance of ‘appropriate’ assumptions for simulating an unknown future to support imaginary models, fail simple logic. As it is impossible to demonstrate the validity of inductive procedures; empirical verification is logically impossible. Claims or assumptions from the past cannot be justified in making assumptions or claims on an unknown future; they cannot be verified only falsified if they are empirically evaluable. Logical positivism and verification were declared dead by the 1960s; yet linger on in CHEERS 22 and the ISPOR/ICER meme. ^19^ Popper claimed the sole responsibility for destroying logical positivism. ^20^

Until Popper’s solution to the problem of induction, there was no firm basis for scientific knowledge; only validation through additional observations or applications. In terms of the logic of statements, no number of observations would allow us to deduce a universal statement as one exception would deny any empirical generalization through validation. ^17^ Post-Popper we could now accept that the laws of science were testable even though unprovable. The result is that the focus in the physical and mature social sciences has been the discovery of new, yet provisional facts. The exception is the CHEERS 22 and ISPOR/ICER health technology assessment where the dominant paradigm is to reject that we have a solution to the problem of induction in favor of claiming to validate imaginary value claims based on prior assumptions. Despite its popularity the approximate information paradigm is a failure; an analytical dead end. If we apply Popper’s demarcation standard between science and non-science, the current health technology paradigm falls squarely into the latter category, encompassing both metaphysics and pseudoscience. None of the thousands (over 10,000) of published assumption driven health technology claims on the future for cost-effectiveness, pricing and access meet the standards for scientific credibility; they are, in Piglucci’s phrase (following Jeremy Bentham) ‘nonsense on stilts’. ^21^ Obviously, no modeled claim on the future can be ranked above other modeled claims. If these are proposed as the basis or at least an input to formulary decisions, then we are in an analytical dead end one that lacks any scientific credibility. All we are left with, if we follow this belief system, is a universe of competing models inventing competing non-evaluable claims expressed in probabilistic sensitivity terms extending into the far future with willing support from academic journals.

### Measurement, attributes and protocols

While understandable, it is surprising that the CHEERS 22 guidance ignores questions of measurement, the importance of reporting single attributes and the role of protocols to provide the framework for evaluating empirical claims linking to real world data. Presumably, if the claims are imaginary then these issues are unimportant. Even so, accurate measure is critical. To paraphrase Lord Kelvin: *If you cannot measure you cannot improve it …. your knowledge is meager and unsatisfactory*. ^22^

Claims for response to therapy must recognize the axioms of fundamental measurement. Following the formalization by Stevens and others in the 1930s and 1940s, scales or levels of evidence used in statistical analyses are classified as nominal, ordinal, interval or ratio. ^23^ Each scale has one or more of the following properties: (i) identity where each value has a unique meaning (nominal scale); (ii) magnitude where values on the scale have an ordered relationship with each other but the distance between each is unknown (ordinal scale); (iii) invariance of comparison where scale units are equal in an ordered relationship with an arbitrary zero (interval scale) and (iv) a true zero (or a universal constant) where no value on the scale can take negative scores (ratio scale). Nominal and ordinal scales only support nonparametric statistics. Interval scales can support addition and subtraction while ratio scales support the additional operations of multiplication and division as they have a true zero. This zero point characteristic means it is meaningful to say that the one object is twice as long as another. Given these limitations, the only acceptable empirically evaluable value claims are those designed for single attributes with interval or ratio properties.

Composite scales in attempting to bundle attributes are disallowed unless they are constructed from ratio scales. One example is body mass index which comprises the two ratio scales of height and weight. This immediately rejects both direct and indirect preference scales as they are bundled health state descriptions capturing a limited list of symptoms, with each symptom scale having ordinal properties (ranked Likert-type scales from no problems to extreme problems). In health technology assessment physical attributes would be expected to have ratio properties, which allow value claims assessment from randomized trials and other real-world data as long as they meet criteria for single attributes. Value claims that attempt to capture latent attributes are more difficult to measure as the instrument for data collection must be designed to generate either ratio or interval properties. Clearly, composite imaginary preference claims are rejected, to include blanket claims for incremental cost-per-QALY, cost-per-QALY thresholds, probabilistic sensitivity analysis and overall cost-effectiveness. ^24^

### Assumptions and false assumptions

One of the intriguing features of the existing approximate information meme is the apparent belief in the choice of realistic assumptions to model an unknown future. The ICER evidence reports, for example, typically provide a list of assumptions and the respective sources as, presumably, the assumptions they think the most relevant. The CHEERS 22 checklist makes no reference to the issue of assumptions and how they might be justified; it merely gives an example of how one might set out the list of one’s preferred assumptions. To refer, for example, to a single study as the source for disease specific modeled ordinal preferences in no way implies that we should expect the next study, utilizing the same design to replicate the results. Indeed, even if there were prior multiple studies yielding the same or similar preference scores we cannot claim or make the statement that the choice has been verified as all preferences (all swans) are confirmed (are white). Irrespective of how many confirmatory observations exist and appeals to probability, we cannot make any claim for the ‘superiority’ or ‘realism’ of one assumption over another; we may believe it, but that is a fact of psychology, not logic. This opens the door to a potential multitude of approximate information models in disease areas driven by competing assumptions each generating its own mix of meaningless non-evaluable claims Any other list of assumptions would be equally valid (or invalid).

We should add to this cornucopia of possible assumptions, the presence of false assumptions. This undercuts the entire ISPOR/ICER modeling methodology. There is no mention of the limitations imposed by the axioms of fundamental measurement on the choice of inputs to populate the simulated economic evaluation. This is a common failing although there have been red flags posted over the past 30 or more years. ^25^^,^^26^ This is of particular interest in the case of quality adjusted life years (QALYs) which are supported by CHEERS 22. ^27^ The QALY is a construct that requires the preference scores to have single attribute, unidimensional bounded ratio properties in a range of zero to unity (0 to 1) with invariance of comparisons (or interval measurement properties which are implicit in a ratio scale). Unfortunately, none of the generic preference scores used in QALY claims has these properties; a point that CHEERS 22 recognizes in pointing to negative preference values but fails to consider their implications. ^28^ For example, the EQ-5D-3L/5L multiattribute instruments are composite bundles of symptoms with only ordinal properties. Claims for the notion of a bundle of symptom driven health related quality of life (HRQoL) fail on the grounds of the axioms of fundamental measurement; composite ordinal structures with no claim to measure response to therapy. They fail to reflect a single latent quality of life attribute and also fail to have a true zero with an upper bound of unity; this is critical as the presence of a true zero means that under no circumstances can there be any negative values. The algorithms applied to create the EQ-5D-3L/5L scores, for example, both produce negative values (health states worse than death). As an example, in a recent US valuation for the EQ-5D-5L, some 20 percent of health states had a negative score. ^29^

The absence of a true zero applies to all direct and indirect preference scales. The reason for this failure is quite clear: no one thought that their preference instrument should be designed by selecting and fitting the required data elements to a latent construct quality of life model, as exemplified in Rasch Measurement Theory (RMT). ^30^^,^^31^ Creating an interval scale for a latent construct such as need fulfillment quality of life through the application of RMT is difficult, although achieved for some 30 disease areas over the past 25 years. ^32^ While RMT can create an interval measure, it is only under certain conditions that it possible to go beyond to a bounded ratio scale. ^33^ Leaders in the field of approximate information appear unaware of the potential role of RMT in instrument development for latent constructs, although it has been applied in education and psychology for the last 60 years. ^34^ Not surprisingly, RMT is not considered in any of the ICER/ISPOR statements of practice standards. Unfortunately, the same ignorance of fundamental measurement holds for the overwhelming majority of disease specific patient reported outcome (PRO) instruments. ^21^^,^^35^

### The failure of approximate information

The commitment to approximate invented value claims rather than hypothesis testing to support value claims has been the mainstay of health technology assessments for over 30 years. The CHEERS 22 contribution to imaginary claims consists of a 28 item checklist which is intended to apply to any form of health technology assessment as guidance for economic evaluation submissions to leading journals in the field. As noted, there is no intent in CHEERS 22 to address the manifest deficiencies of the current health technology meme. In summary, these are:

• A failure to address the fundamental difference between science and non-science: all value claims must apply to a single attribute whether in physical or latent terms where the claim is credible, empirically evaluable and replicable. All claims from economic evaluations must be empirically evaluable, ideally supported by a protocol to detail the assessment process. If not, the claim must be rejected.
• A failure to recognize the limitations imposed by the axioms of fundamental measurement where all value claims must meet not ordinal but ratio or at least interval measurement standards.
• A failure to recognize that if a value claim is to meet ratio or interval measurement standards, instruments must be designed to have these required measurement properties.
• A failure to appreciate that generic multiattribute preference scores such as the EQ-5D-3L/5L have only composite ordinal properties and, as such, cannot capture response to therapy (they lack the ability to support standard arithmetic operations such as multiplication, division, additional and subtraction). Put simply they lack a true zero as the algorithms produce negative values; apart from their lack of dimensional homogeneity and construct validity.
• A failure to accept that the quality adjusted life year (QALY) is an impossible mathematical construct if the applicable preference score has ordinal properties and relates to multiple attributes; a composite or bundle of symptoms that each fail to have the requisite measurement property.
• A failure to recognize the adverse implications of a mathematically impossible QALY in attempts to make modeled incremental lifetime cost-effectiveness claims and the application of equally mathematically impossible cost-per-QALY thresholds.
• A failure to recognize Hume’s problem where basing assumptions for future claims on past observations is logically indefensible: an assumption is an assumption, there can be no preference for one assumption regarding an unknown future over another.
• A failure to admit that lifetime assumption driven simulations are redundant as a basis for any decision making as they have no credibility, typically based on imaginary QALYs and assumed future costs leading to tools such as probabilistic sensitivity analysis and non-evaluable claims for cost-effectiveness.
• A failure to admit that as the imaginary modeled lifetime value claims are driven entirely by assumptions regarding an unknown future, there is ample scope to produce any number of competing value claims with no basis for judging the merits of one set of imaginary claims over another.
• A failure to recognize that with the manifest deficiencies that characterize the approximate information assumption driven lifetime modeling, statements that a product is cost effective are meaningless as the door is open to number of competing cost-effectiveness claims with each one failing the required standards.

### Abandoning models

The implications of this assessment should be quite clear: unless a model, irrespective of whether it is a short term model built on a clinical trial or an extrapolated from a trial produces empirically evaluable claims it must be rejected. There are no exceptions. Applying these criteria to the thousands of approximate information models published over last 30 years in leading academic journals means that the overwhelming majority should be withdrawn. Given this we can interpret CHEERS 22 as, hopefully, the last attempt to justify a flawed approximate information methodology. A more substantive question is whether we need models in the first place. If the focus is on single attributes as value claims then modeling is irrelevant. The focus should be on evidence gaps, the discovery of new, yet provisional facts, and the continuing evaluation of therapy claims as part of disease area and therapeutic class reviews. This is not to deny the development of models where it is possible, if for example the claim is falsified, to review the assumptions driving the model and return with an ‘improved’ version.

### Formulary decision making

Given the irrelevance of the approximate information meme as providing meaningful and evaluable inputs to formulary decision making, the next question is: what form should a formulary submission take? The answer is not difficult and has, in fact, been addressed some years ago, notably in terms of protocol standards (PROST) for evaluating claims (but not referenced by CHEERS 22). ^2^^,^^3^ The required standards for value claims for response to therapy that are relevant for formulary decisions are:

• All value claims must refer to single attributes that meet the demarcation standards for normal science: they must be credible, evaluable and replicable
• All value claims must be consistent with the limitations imposed by the axioms of fundamental measurement: they must meet interval or ratio standards

Where a submission is to be made the relevant value claims that meet required measurement standards should be at the discretion of the formulary committee and appropriate to the target patient population in the disease area. Generic preference instruments should be put to one side while, at the same time, disease specific PROs should be assessed for their measurement properties. It is worth noting that the only formulary guidance that subscribes to the approximate information paradigm in the US is version 4.1 of the Academy of Managed Care Pharmacy (AMCP) Format for Formulary Submissions. ^36^ It is unclear how widely it is applied by formulary committees including managed care organizations and pharmacy benefit managers given its acceptance of the approximate information meme. In contrast, the approximate information meme is rejected by Version 3.0 of the Minnesota formulary guidelines. ^37^ These guidelines recognize the need to accept the standards of normal science for falsification and the limitations of fundamental measurement. Assumption driven simulations have no role in value claims; neither do blanket claims for cost utility, cost-effectiveness and social pricing. Instead the Minnesota guidelines subscribe to the standards presented here for value claims.

### Questions a formulary committee should ask

The formulary committee should have prepared a minimum list of attributes that are required to support a formulary decision. These can be expressed in clinical terms, typically as ratio measures, PROs that meet required single attribute measurement standards, resource utilization (not costs) and compliance. Following from the earlier PROST guidance for reviewing submissions, formulary committees should address the following questions:

• Are the submitted value claims consistent with the claims that the formulary committee has determined are required and appropriate for the indicated product in the target patient population?
• Are the submitted value claims presented in a form that is credible, evaluable and replicable?
• Are the submitted value claims for single attributes that have either ratio or interval measurement properties?
• Are the measurement properties detailed for each value claim?
• If the value claims are comparative, do the chosen comparators reporting meet the required measurement standards?
• Are value claims based on pivotal phase 3 trials to be replicated?
• Are all value claims capable of being empirically evaluated in a meaningful time frame?
• Has a protocol been provided to demonstrate how each value claim is to be evaluated?
• Is it proposed how the value claims might be integrated into a long term research strategy to support the discovery of new facts and to support ongoing disease area and therapeutic class reviews for the indicated target patient population?

## Conclusions

The purpose of this commentary has been to demonstrate the manifest deficiencies of the current approximate information meme and the questionable support given to imaginary modeled claims by the CHEERS 22 reporting guidance. At best, the CHEERS 22 guidance should be either withdrawn or brought into line with the required standards of normal science; this is unlikely. It is easy to speculate, but a relevant question is why CHEERS 22 failed to address any of the substantive criticisms of its belief system that have been raised over a number of years and is well documented and accessible. The ISPOR house journal, *Value in Health*, is perfectly aware of these criticisms but ISPOR has never attempted to respond to them. ^38^ Yet, on a more positive note, we know the key elements for a new start in technology assessment to support formulary evaluations. It marks a sea change from approximate non-evaluable claims, abandoning lifetime simulation models, to those claims that meet the measurement and associated standards of normal science. We must extract ourselves from the ISPOR rabbit hole that is nothing more than an analytical dead end. Continuing denial or oversight of the required standards does nothing to address patient, physician and health system needs.

As the typical simulation model uses generic ordinal preference scores to create mathematically impossible QALYs, utilizing these composite ordinal preference scores to model value claims expressed in incremental cost-per-QALY terms, the entire exercise and created value claims are meaningless. ICER has tried to argue that the generic ordinal scores are actually ratio measures in disguise with bounded ratio properties; this is a patently absurd claim. ^39^^,^^40^ What is not addressed is a key question: if you want a single attribute bounded ratio measure or at least an interval measure to measure response to therapy then it has to be designed from the start. This obviously did not happen.

Challenging an established meme is not an easy task; fortunately, all the elements of a new start paradigm as a recommended framework for formulary submissions and claims assessment have been addressed in this commentary. It is quite straight forward in respect of: (i) the value claim attributes required to be addressed for target populations in disease areas; (ii) the measurement standards required for those attributes; and (iii) the protocol for evaluating and reporting on attribute value claims. We should abandon any attempt to develop and present imaginary modeled claims and, indeed, blanket claims for cost-effectiveness. The new start paradigm focuses on information needs and assessing those needs, with the emphasis on the standards of normal science and fundamental measurement as part of an ongoing process of disease area and therapeutic class reviews. We have an obligation to report on the discovery of new, yet provisional, value claims rather than a continuing focus on imaginary modeled claims.

## Data availability

No data are associated with this article.
