# Supplementary material for: Nothing to Cheer About: Endorsing  Imaginary Economic Evaluations and Value Claims with CHEERS 22
Source: F1000Res. 2022 Feb 28;11:248. [Version 1] doi: 10.12688/f1000research.109389.1 (PMC8994082; doi:10.12688/f1000research.109389.1)
Supplement: Peer review history

# Peer review: Reviewer response for version 1

**DOI:** 10.5256/f1000research.120883.r125724

**Published:** 2022-04-08

**Authors:** Jonathan Belsey

**Affiliations:** 1 JB Medical Ltd, Sudbury, UK

The primary objective of this paper is to make a case that the recently published Consolidated Health Economic Evaluation Reporting Standards 2022 (CHEERS 22) checklist is flawed, in that it fails to address the fundamental limitations of the vast majority of cost utility analyses, based as they are on a metric that fails to meet the required statistical standards that make them fit for purpose. The author has comprehensively addressed this issue in previous publications and makes a compelling, well evidenced case for the mathematical shortcomings of the QALY as an appropriate means of quantifying the utility component of a cost utility analysis.

 I do not propose to re-visit the authors arguments in detail in this review but suffice to say that it is difficult to refute them on any rational scientific grounds. The problem is, of course, that conventionally accepted practice – even in a nominally mathematically based discipline like health technology appraisal – is rarely based on solid science. Anybody who has had dealings with health technology appraisal bodies worldwide will understand that the usual role of health economic analysis is to provide quantitative support for prior expectations and where it fails to do this, the modelling approach is altered to ensure that it does. The very fact that such a strategy is both commonplace and effective should alert the independent thinker to the possibility that the underlying methodology is flawed – a robust mathematical approach should be robust to assumptions and manipulation, whereas mainstream health economic analysis is anything but.

 But herein lies the problem. The author has pointed out – not for the first time – that the emperor has no clothes. Unfortunately there are health care prioritisation systems set up throughout the world that depend utterly on the existence of a naked emperor. There is too much academic, commercial and political interest vested in the status quo to allow lone voices – however well argued – to disrupt the narrative.

 I absolutely agree with the commentary that the author has written and I am also convinced that the alternative structure that he proposes would yield more reliable results. Sadly, however, even though the individuals involved in HTA may privately acknowledge the accuracy of his arguments, the chances that they will change is negligible. Their belief is that the current process may be fundamentally unreliable, but it is comfortable and familiar and sufficiently complex that 99% of people will fail to understand the flaw at its core. Sadly, I believe it will be business as usual for the foreseeable future.

Is the topic of the opinion article discussed accurately in the context of the current literature?

Yes

Are arguments sufficiently supported by evidence from the published literature?

Yes

Are all factual statements correct and adequately supported by citations?

Yes

Are the conclusions drawn balanced and justified on the basis of the presented arguments?

Yes

Reviewer Expertise:

Health economics & Health Technology Assessment

I confirm that I have read this submission and believe that I have an appropriate level of expertise to confirm that it is of an acceptable scientific standard.

# Peer review: Reviewer response for version 1

**DOI:** 10.5256/f1000research.120883.r125721

**Published:** 2022-03-29

**Authors:** Uwe Albrecht

**Affiliations:** 1 Director, Mediconomics, Hanover, Germany

The critical elements of this article focus on the principles of research and the mathematical models in this context of health research. The first argument is related to choosing appropriate scales to measure the required data as the outcome of interventions. Current literature highlights and supports that the approaches used over the decades clearly lack a scientifically valid model to capture essential data, and consequently, results are questionable. This is especially true as various questionnaires and scales result in misleading numerical values, which are mathematically wrong. The second argument is related to correct scientific research, again strongly supported by current literature. Finally, it is convincing that research must be reproducible and based on valid measurement instruments.

 The citations for this article allow for a complete overview of this specific field of research and evaluation of results. Clinical studies are frequently reported in the context of a complex compilation of various data. This ranges from measurements of laboratory data that present valid biological values at one point in time to a combination of subjective outcomes reported by the patient, for instance, pain or well-being. The crucial point is not to mix data reported as primary outcomes in mathematical terms with data based on perceptions that are reported on scientifically questionable scales. The view of the factual statement is convincing and clearly understandable for the reader.

 The arguments of this article are based on and supported by public literature cited and listed in the references. There is ample evidence that the author has published on this topic in many excellent journals and monographs before and has reviewed the situation of outcomes research in-depth. It is essential to consider the arguments on the background of the importance of the task which is to be fulfilled. There are serious questions that policymakers should consider regards the methodology and the resulting impact on patients and health care systems. Misleading approaches to postulate the assumed benefit of specific interventions may result in a burden to the health care system and incorrect as well as possible dangerous recommendations. It is convincing to highlight that facts must be the fundamental guidance for decision takers rather than data composed of validated but not targeted invented scales - or even worse - statistical models, that fail to reflect excellent and reproducible outcomes. Therefore, it is mandatory to carefully consider this article's arguments in accepting or rejecting health research results.

 The conclusions in the article are well presented and display a clear picture of arguments that are based on the observation and interpretation of approaches to generate data for policy-making and decision-making in the health care sector. The justification is based on a concise explanation of proper scientific research and the resulting guidance on which the scientific community has relied since modern research started. It mirror-images the understanding of practical research, which reports data generated in a trial context and elements associated with the impact of an intervention. Overall, it is crucial to recognize that not every complex or straightforward modelling is relevant, primarily if no apparent reason exists for the choice of the assumptions.

Is the topic of the opinion article discussed accurately in the context of the current literature?

Yes

Are arguments sufficiently supported by evidence from the published literature?

Yes

Are all factual statements correct and adequately supported by citations?

Yes

Are the conclusions drawn balanced and justified on the basis of the presented arguments?

Yes

Reviewer Expertise:

NA

I confirm that I have read this submission and believe that I have an appropriate level of expertise to confirm that it is of an acceptable scientific standard.
